# Reconstruction of Chronic Soft Tissue Mallet Fingers: Outcomes of Step-Plasty vs. Purse-String Suture

**DOI:** 10.3390/jfmk9030144

**Published:** 2024-08-24

**Authors:** Wolfram Demmer, Andreas Frick, Rüdiger G. H. Baumeister, Elisabeth Haas-Lützenberger, Nikolaus Thierfelder, Sinan Mert, Denis Ehrl, Riccardo Giunta, Christiane G. Stäuble

**Affiliations:** 1 Department of Hand, Plastic and Aesthetic Surgery, LMU Klinikum, Ziemssenstraße 5, 80336 Munich, Germanyriccardo.giunta@med.uni-muenchen.de (R.G.); 2 Lymphology Unit, Urologische Klinik Muenchen Planegg, 82152 Planegg, Germany; 3 Department of Plastic, Reconstructive and Hand Surgery, Burn Centre for Severe Burn Injuries, Nuremberg Clinics, University Hospital Paracelsus Medical University, 90419 Nuremberg, Germany; 4 Klinik für Anaesthesiologie, Klinikum rechts der Isar, Technische Universität München, 81675 Munich, Germany

**Keywords:** extensor tendon, mallet finger, subcutaneous extensor tendon rupture, purse-string suture, swan neck deformity, drop finger, Crawford’s criteria, Levante’s criteria

## Abstract

After failed conservative therapy or in the absence of any intervention, a rupture of the digital subcutaneous extensor tendon at the distal interphalangeal (DIP) joint, known as mallet finger, may lead to a chronic extension deficit due to excessive scarring and tendon elongation. Various surgical techniques to restore the extension of the distal phalanx have been proposed, but an optimal approach has not yet been established. To tighten the extensor tendon, a purse-string suture can be applied. Although it has shown efficacy, it can result in significant bulging and scar formation. Using the “abbreviato” technique, the elongated part of the extensor tendon is excised, and the tendon is re-sutured. Also, tenodesis has been described, particularly in pediatric cases. In this retrospective follow-up study, we aimed to investigate if the step-plasty procedure previously described by Baumeister provides comparable, if not superior, functional and aesthetic outcomes compared to existing techniques for patients with chronic mallet finger. In this retrospective study, a consecutive series of 68 patients with chronic mallet fingers was enrolled. Patients were treated surgically using step-plasty of the respective extensor tendon. After skin incision and tenolysis, the elongated extensor tendon was incised in a Z-like fashion and stepwise resected in the transverse portion of the Z. The functional and aesthetic effects of this step-plasty technique were compared with results of 44 patients previously treated using purse-string sutures of the extensor tendon and evaluated using Crawford’s and Levante’s criteria. In all patients undergoing the step-plasty procedure, the extension deficit was significantly reduced from an average of 42 degrees preoperatively to 11 degrees postoperatively. In contrast, the control group treated by purse-string sutures showed a slightly higher postoperative extension deficit of 15 degrees. According to Levante’s criteria, the results of our step-plasty procedure were significantly better than those achieved with purse-string sutures. Our study demonstrated that the treatment of older or chronic subcutaneous extensor tendon ruptures using the step-plasty technique led to a significant reduction in extension deficits. According to Levante’s criteria, the postoperative outcome was significantly better in comparison to the purse-string suture technique. Additionally, no skin resection was required to improve the extension capability of the distal finger joint, compared to established surgical procedures.

## 1. Introduction

Mallet finger is an injury affecting the extensor tendon at the distal interphalangeal (DIP) joint of a finger. Mallet finger injuries most frequently affect middle-aged men and older women. It is estimated that the incidence of mallet finger injuries is approximately 9.89 per 100,000, commonly occurring in the long, ring, and small fingers. These injuries are often sustained during work or sports activities, especially ball sports. The injury can result from varying degrees of trauma, ranging from significant to relatively minor [1,2,3,4]. The injury can be limited to a subcutaneous rupture of the extensor tendon or an avulsion of the tendon from the distal phalanx, or it can involve a bony avulsion of the tendon insertion at the distal phalanx [1,2].

The acute subcutaneous or bony avulsion of the extensor tendon is classified according to Doyle into the following four types: Type 1 (closed injury, with or without a small dorsal avulsion fracture), Type 2 (open injury with laceration of the tendon), Type 3 (open injury with loss of skin, subcutaneous cover, and tendon substance), and Type 4 (A–C) mallet fracture of the distal phalanx. Depending on the type, different treatments are recommended [3]. In acute mallet finger Type 1 injuries, the DIP joint is typically splinted in full extension for 6 to 8 weeks. For Type 2 and 3 injuries, treatment recommendations include the debridement and suturing of the tendon. The treatment of bony fractures in Type 4 injuries remains controversial. Some authors recommend purely conservative management, even for displaced injuries [4]. Nevertheless, many surgeons advocate for surgical intervention for mallet fractures that involve more than one-third of the articular surface or for fractures with associated DIP joint subluxation. Various surgical techniques have been described, which all share the common approach of temporary transfixion of the DIP joint using K-wire [1]. If mallet injuries are not treated or are inadequately managed, patients may develop chronic mallet fingers. Over a period of three to four weeks, or sometimes even longer, they may suffer from a drooping fingertip with an extension deficit, a swan neck deformity, or dissatisfaction with the appearance of the finger [5]. The reason for this is usually an elongated scar in the area in which the extensor tendon has ruptured [1]. This condition can hinder their normal work and recreational activities. Various surgical techniques are described to reduce the functional and aesthetical impairment caused by an extension deficit of the distal finger joint [1,6]. A chronic mallet finger caused by an untreated or inadequately treated extensor tendon injury can still be treated conservatively with splinting. Different authors suggest a time frame ranging from several weeks to up to six months since the initial injury [7,8,9,10,11]. Non-surgical approaches to treating chronic mallet finger can be very time-consuming, often requiring the affected finger to be splinted for up to 10 weeks. This prolonged immobilization can lead to subsequent stiffness of the DIP joint, impairing the patient’s ability to perform daily work and activities. According to the literature, some authors indicate surgery when the extension deficit becomes symptomatic, with more than 10 to 40 degrees [6,12]. Surgical procedures for chronic mallet finger deformities are designed to stabilize the DIP joint and enhance active extension [13]. An older but still commonly used technique is the purse-string suture introduced by George et al. With this technique, the elongated tendon is grasped by a circular, continuous, running subcutaneous suture. The tightening of the suture leads to a bulging and thus shortening of the elongated scar tissue [14]. Other examples of surgical treatment include terminal extensor tendon shortening without excision of the scar, the so-called “abbrevatio” procedure [15], and tenodermodesis, especially in children between 1½ and 18 years of age [16,17]. In advanced cases presenting with a swan neck deformity, oblique retinacular ligament reconstruction may be necessary [18]. Additionally, arthrodesis of the DIP joint may be considered as a last resort to achieve functional stability [19].

Only a thin subcutaneous layer covers the distal extensor region of the long finger (extensor tendons zones 1–2) [20]. Tendon shortening procedures, such as a purse-string suture causing a pleat of the extensor tendon or the “abbrevatio” technique without excision of the elongated scar tissue, can add additional subcutaneous bulk to the area [1,21,22]. To reduce the thickness after reconstruction or tightening of the extensor tendon and address these impairing factors, Baumeister modified the surgical technique. By transecting the tendinous scar step-by-step and excising the superficial scar tissue, the adjacent undamaged tendon is sutured in a Z-shaped manner [23]. The previous results of this “step-plasty” technique for tightening extensor tendons in chronic mallet fingers suggested improved postoperative outcomes regarding the extension deficit [23]. In this study, the postoperative outcome of the “step-plasty” technique was compared to the results of well-established purse-string sutures in chronic mallet fingers. We investigated the postoperative outcomes following the step-plasty reconstruction of the extensor tendons in zones 1–2 in a larger cohort. Simultaneously, the results were compared with a group of patients who were treated with purse-string sutures. The aim of this study was to determine whether there is a significant difference in the postoperative results between the step-plasty method presented here and the well-established purse-string technique, focusing on the functional and aesthetic outcomes of both techniques.

## 2. Materials and Methods

### 2.1. Study Design

In this retrospective study, we evaluated the pre- and postoperative status of patients suffering from chronic soft tissue mallet fingers treated by step-plasty and purse-string sutures, respectively.

Patients suffering from chronic mallet finger after initial closed subcutaneous extensor tendon rupture without bony involvement (Doyle Type 1) were included in this study. Chronic mallet finger was defined as a persistent extension deficit of the DIP joint of more than 20° after an unsuccessful treatment attempt with a Stack splint for at least 8 weeks, followed by night splinting for 4 weeks. Joint stiffness was treated with physiotherapy beforehand. All patients participating were over 18 years old, desired surgical treatment after thorough information about alternative treatment methods, and agreed to participate in this study. Patients with open injuries who were treated with primary tendon repair were excluded (Doyle Type 2 and 3). All patients received a preoperative X-ray. Patients with bony lesions or joint disorders, such as degenerative joint disease or detected subluxation, were excluded from this study (Doyle Type 4). Patients with chronic mallet fingers treated by other methods, such as tenodermodesis, were not included in this retrospective study. Data were collected for nearly two decades (1991 to 2011) from all patients who underwent a step-plasty reconstruction of the extensor tendon and met the inclusion criteria. At the first preoperative and last postoperative examinations of the patients, the active range of motion (ROM) of the affected finger was prospectively documented using the neutral 0 method for later evaluation. The control group consisted of data from patients treated with traditional purse-string sutures, retrospectively retrieved from our hospital’s documentation. These data have previously been used as control data in the initial article presenting the step-plasty by Baumeister [23]. Although the control group was considerably smaller, there were no relevant differences in its composition with respect to age or the interval between the accident and surgery compared to the study group.

All procedures were conducted by two trained hand surgeons from our department with the assistance of surgical interns [24]. 

The maximal active range of motion was measured from the neutral position while the patient was sitting upright with arms at their sides and fingers fully extended. Complete passive extension and flexion were also assessed. Extension and flexion of the fingers were measured with a standard hand goniometer using the neutral 0 method of the Association for the Study of Internal Fixation (AO) [25]. The indication for surgery was established at an extension lag greater than 20°, measured using the neutral 0 method.

The functional outcomes were assessed using Crawford’s criteria and Levante’s criteria [26,27] (Table 1). 

In addition to the pre- and postoperative range of motion, epidemiological data, such as gender, age of the patients at the time of surgery, and the interval duration since the operation, were collected and evaluated. A sample size calculation was performed before evaluating the retrospective data. The number of patients per group allowed for a two-sided unpaired t-test with an effect size of d = 0.6, an alpha level of 0.05, and a power of 0.8 to detect medium effect sizes. To determine statistically significant differences, paired and independent samples t-tests, as well as Pearson’s Chi-square test, were used. A *p*-value of less than 0.05 was considered statistically significant.

Ethical approval for this study was obtained from the Ethics Committee of the LMU Munich (Project No.: 24-0164).

### 2.2. Surgical Procedures

**Figure 1:**
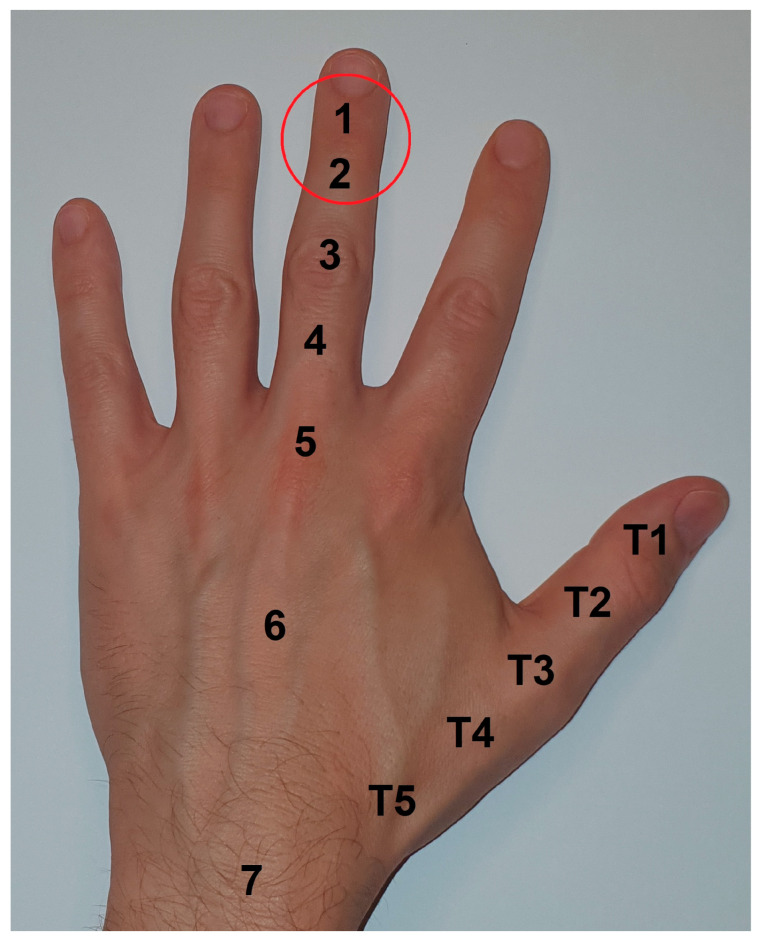
Classification of extensor tendon zones according to Kleinert and Verdan [20]; surgical site: zone 1–2, extensor tendon insertion and DIP joint.

Chronic mallet finger as a complication of old subcutaneous extensor tendon avulsions (Doyle Type 1) occurs in zones 1–2 according to Kleinert and Verdan. Figure 1 shows the surgical site in relation to the extensor tendon zones.

**Figure 2:**
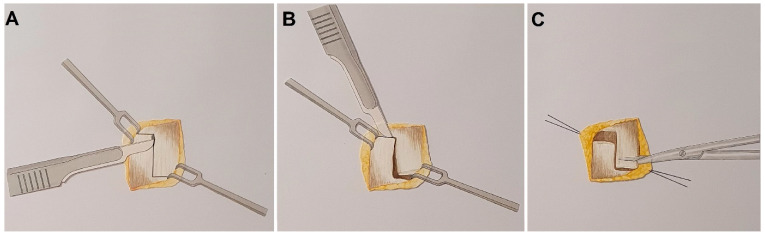
(**A**,**B**): Stepwise incision of the tendon and resection of elongated scar/tendon tissue (**C**): Excision of scar tissue.

For this study, a standardized surgical approach was used; the skin was incised at the dorsum of the finger in the area of the distal joint in an s-shaped pattern. The underlying tendon was exposed, and surrounding scar tissue was meticulously removed. The tendinous scar and adjacent tendon were then carefully incised in a stepwise manner, with approximately 2–3 mm of transversely resected scar/tendon tissue at the incision site (Figure 2).

**Figure 3:**
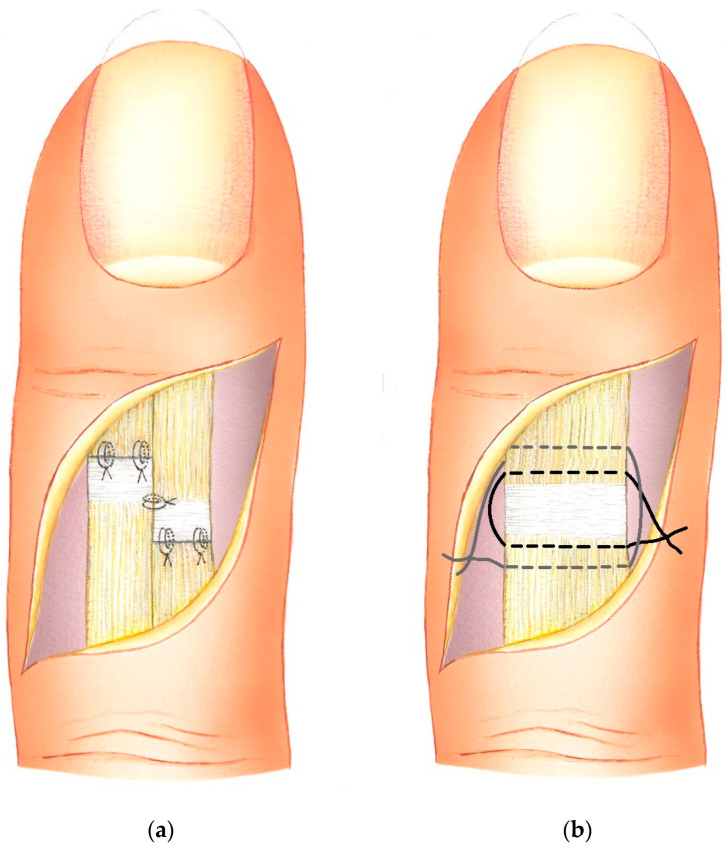
(**a**) Sequential suturing of the step-cut incised extensor tendon in zones 1–2; (**b**) double purse-string suture of elongated scar tissue of extensor tendon in zones 1–2.

The DIP joint is brought into extension and stabilized in a neutral position using a transarticular K-wire. The step-cut tendon segments were first sutured side-to-side centrally along the longitudinally incised parts using 4–0 long-lasting absorbable sutures. In the transverse tendon segments, where tendon/scar surplus war resected, a figure-of-eight pattern with the same 4–0 sutures was performed (Figure 3a). Through the sequential suturing technique, the tension on the tendon ends is minimized during the placement of the transverse sutures.

In the control group of patients treated using purse-string plasty, a curved incision of the dorsum of the finger was conducted. The visible scar tissue causing the pathological elongation of the extensor tendon was carefully released from its underlying structures without opening the distal interphalangeal joint. Two purse-string sutures (3–0 long-lasting absorbable) were used to tighten the scar tissue, causing a bulge at the same time (Figure 3b). Tendon traction places the distal phalanx in an overstretched position, which is maintained with a K-wire through the DIP for five weeks.

### 2.3. Postoperative Care

After surgery, fingers were splinted with a palmar cast for three weeks, and afterward, they were treated with a Stack splint for another one to two weeks up to the extraction of the K-wire. In total, the respective finger was immobilized for approximately five weeks after surgery. After removal of the K-wire used for transfixion of the DIP joint, patients were instructed to mobilize their fingers independently within a pain-free range for one week, avoiding weight-bearing activities to prevent re-rupture due to early overload. Subsequently, physiotherapy was initiated. Upon completion of physiotherapy, all patients were scheduled for a follow-up examination.

## 3. Results

A period of approximately two decades was examined retrospectively, reaching from 1991 to 2011. All patients included in this study exhibited symptomatic mallet fingers, complained of interference with their normal work and recreational activities, which were sometimes associated with pain, or were dissatisfied with the appearance of the affected digit. The extension deficit of the concerned DIP joint was passively correctable in all patients prior to surgery.

During the period observed, 68 patients received a step-plasty for extension lag due to chronic mallet finger. The group consisted of 40 women (58.8%) and 28 men (41.2%), with a mean age of 48 (±14 SD) years. The retrospective control group, treated with tendon shortening using the well-established purse-string suture technique, consisted of 44 patients, 25 (57%) women and 19 (43%) men with a mean age of 43 (±12 SD) years [23].

In the patient group treated with step-plasty, the time from the initial injury through the conservative therapy attempt to revision surgery of the chronic mallet finger averaged 2.7 (±0.8 SD) months. In the control group treated with purse-string sutures, this interval was 2.9 (±1.2 SD) months. There was no statistically significant difference in the preoperative extension deficit between the step-plasty and purse-string groups, with 42° (±11 SD) and 40° (±11 SD), respectively.

In patients treated using step-plasty, the mean preoperative extension deficit significantly decreased by an average of 31°, improving from 42° (±11 SD) preoperatively to 11° (±9°) at the follow-up examination 4 (±6 SD) months after surgery (*p* < 0.05). The postoperative flexion deficit of the PIP joint was 9° (±9 SD). According to Crawford’s criteria, 65% of patients treated with step-plasty achieved excellent and good results [26] (Table 2). Using Levante’s criteria, 67% showed very good and good results [27] (Table 3).

The control group treated with purse-string sutures also exhibited a significant reduction in extension deficit at the DIP joint. The preoperative extension deficit improved from 40° (±11 SD) to 15° (±11 SD) in the follow-up examination after an average of 3 (±2 SD) months (*p* < 0.05).

The postoperative flexion lag was 11° (±19°). According to Crawford’s criteria and Levante’s criteria, 38% of the treated patients achieved excellent and good results [26,27] (Table 2 and Table 3).

There was no difference in the total range of motion (ROM) of the DIP joints, which measured 60° (±3 SD).

When comparing both techniques using Crawford’s and Levante’s criteria, respectively, patients treated with step-plasty showed superior postoperative results. According to Levante’s criteria, the postoperative outcome of the step-plasty procedure was significantly better than that of the purse-string suture (*p* = 0.049, *p* = 0.018).

### Complications

Following the step-plasty procedures, two patients experienced K-wire infections. One patient recovered without complications, while the other ultimately developed ankylosis of the distal interphalangeal joint despite adequate systemic and local antibiotic therapy. One patient treated with a purse-string suture exhibited signs of complex regional pain syndrome two weeks after the K-wire removal, occurring seven weeks after the initial surgery. Within four weeks, these symptoms resolved completely with no residual extension deficit. In both procedures, temporary arthrodesis and postoperative immobilization were performed. Therefore, no causal relationship with the extensor tendon injury can be assumed.

## 4. Discussion

In this study, the step-plasty technique, a modification to the Linds technique by Baumeister, was evaluated [23]. We could demonstrate that the treatment of chronic subcutaneous extensor tendon rupture with step-plasty significantly improves postoperative extension deficit. Additionally, a significant improvement in extension deficit was observed in the control group treated with purse-string sutures according to George. Both Crawford’s and Levante’s criteria are used to classify postoperative outcomes in soft tissue mallet finger cases. A direct comparison shows that the step-plasty technique outperforms purse-string sutures according to both classifications, with Levante’s criteria indicating a significantly better result. Since Levante tolerates an extension deficit of 5° for a very good outcome and up to 15° for a good postoperative result, this more lenient classification may explain the more favorable results of the step-plasty technique according to Levante’s criteria compared to Crawford’s criteria.

An important consideration is the extent of scar resection. The physiological excursion of the tendon in its distal part is less than 5 mm; resection should ideally not exceed 2–3 mm to minimize the risk of an impaired DIP flexion [28,29]. While Ulusoy and colleagues [30] found the remaining distal tendon stump in soft tissue mallet finger cases not suitable for suture, we consistently found suturable tissue and were able to readapt the tendon stumps in a stepwise manner. In our series, we did not observe any re-ruptures. Some re-ruptures may have been missed during our follow-up period, which was short in some cases.

Another potential postoperative issue after the surgical treatment of chronic mallet fingers is a limitation of flexion at the DIP joint. In our study, the mean flexion deficit was 9° (±9 SD) following the step-plasty procedure, compared to 11° (±19 SD) following purse-string sutures. The mean range of motion was 60° ± 3 for both groups, which is better, as the minimum estimated by Hume and colleagues as a functional range of motion of the DIP joint is 39° for performing daily activities [31].

The optimal approach for treating subcutaneous extensor tendon ruptures remains a subject of controversial debate [32]. Our study showed that, in indicated cases of surgical therapy for chronic mallet fingers, the step-plasty technique for shortening the extensor tendon is an equally good approach as the well-established techniques like the purse-string technique.

We believe that this step-plasty technique also offers aesthetic advantages over other methods, although these aspects were not quantitatively measured in our current study.

The selected approach of comparing the step-cut step-plasty technique with a retrospective control group is conducted similarly to most studies on the surgical treatment of chronic extensor tendon rupture. Nevertheless, the retrospective nature of this study, especially concerning the control group treated using the purse-string suture, is one of its limitations. Another limitation is the comparison of the step-plasty technique only against one other established surgical technique and not against other surgical or conservative treatments. Although both sample groups do not differ significantly in their composition, the overall relatively small sample size and the difference in the number of participants between the step-plasty and control groups present another limitation. Both techniques examined showed significant improvements in postoperative outcomes compared to the preoperative condition. A significant difference was observed only in the outcome criteria according to Levante. Further prospective studies comparing step-plasty with alternative suture techniques are desirable to enhance our understanding and treatment of chronic mallet injuries.

## 5. Conclusions

A significant reduction in extension deficit can be achieved in older or chronic mallet finger cases using the step-cut technique. According to Levante’s criteria, the postoperative outcome is significantly better compared to treatment with the established purse-string suture technique. Additionally, no skin resection was required to improve the extension capability of the distal finger joint, compared to established surgical procedures. The step-plasty technique is a valuable and reliable therapy for chronic mallet finger.

## Figures and Tables

**Table 1:** Functional outcomes assessment using Crawford’s criteria and Levante’s criteria. (ED = extension deficit, CAM = complete active mobility).

Crawfords’s Evaluation Criteria (1984) [26]					
Grade	Excellent	Good	Fair	Poor	
Description	Full DIP joint extension, full flexion, no pain	0–10 degrees of extension deficit, full, flexion, no pain	10–25 degrees of extension deficit, any flexion loss, no pain	More than 25 degrees of extension deficit, or persistent pain	
**Levantes’s Evaluation Criteria (2003)** [27]					
Grade	Very good	Good	Middle	Bad	Failure
Description	ED < 5°, CAM > 60°	ED < 15°, CAM > 50°	ED < 25°, CAM > 40°	ED < 35°, CAM > 30°	ED < 40°, CAM < 20°

**Table 2:** Outcome of soft tissue mallet fingers treated operatively with the step-plasty (n = 68) and purse-string (*n* = 44) techniques according to Crawford’s criteria.

Operative Procedure	Excellent	Good	Fair	Poor	Complications
step-plasty	10 24%	17 41%	12 28%	3 7%	2 (osteitis) 3%.
purse-string	8 22%	6 16%	16 43%	7 19%	1 (dystrophic reaction)

**Table 3:** Outcome of soft tissue mallet fingers treated operatively with the step-plasty (n = 68) and purse-string (n = 44) techniques according to Levante’s criteria.

Operative Procedure	Very Good	Good	Middle	Bad	Failure
step-plasty	10 24%	18 42%	7 17%	7 17%	- -
purse-string	7 19%	7 19%	14 37%	6 17%	3 8%

## Data Availability

The original contributions presented in the study are included in the article, further inquiries can be directed to the corresponding author.
